# Encryptomics: how Aristotle's causality system can be applied to new molecule discovery

**DOI:** 10.1186/1753-6561-8-S4-O27

**Published:** 2014-10-01

**Authors:** Carlos Bloch Junior

**Affiliations:** 1 Laboratório de Espectrometria de Massa - Embrapa Recursos Genéticos e Biotecnologia, Embrapa, Brasília - DF, Brazil

## 

Aristotle (384 BC-322BC), the Greek philosopher formulated the concept of the Four Causes (material, formal, final and efficient causes), which in his view could explain how everything came about in the universe but its beginnings, called by him as the First Cause or the Uncaused Cause. Presently, modern Physics have a more elaborated view of the same principle considering that four discrete forces govern the interactions of matter in the known universe: gravity, electromagnetism, weak and strong nuclear force. As for the origins of the universe, we haven't made much conceptual progress since the Stageirian's work centuries ago.

Inspired by the Aristotelian Causality perceptiveness, its applications to chemical and biological systems, a novel screening strategy was conceived to find new biologically active molecules encrypted inside "parent macromolecules". The method [1] relays on *in silico *descriptors of a given activity (antimicrobial, opioid, inhibitors of enzyme activities), solid-phase peptide synthesis, mass spectrometry and NMR structural analysis, *in vitro *and *in vivo *assays [2].
